# mRNA expression profiles of primary high-grade central osteosarcoma are preserved in cell lines and xenografts

**DOI:** 10.1186/1755-8794-4-66

**Published:** 2011-09-20

**Authors:** Marieke L Kuijjer, Heidi M Namløs, Esther I Hauben, Isidro Machado, Stine H Kresse, Massimo Serra, Antonio Llombart-Bosch, Pancras CW Hogendoorn, Leonardo A Meza-Zepeda, Ola Myklebost, Anne-Marie Cleton-Jansen

**Affiliations:** 1 Department of Pathology, Leiden University Medical Center, Albinusdreef 2, 2300RC Leiden, the Netherlands; 2 Department of Tumor Biology, the Norwegian Radium Hospital, Oslo University Hospital, Montebello, 0310 Oslo, Norway; 3 Department of Pathology, University Hospitals Leuven, Minderbroedersstraat 12, 3000 Leuven, Belgium; 4 Department of Pathology, Valencia University, Av de Vicente Blasco Ibáñez 17, 46010 Valencia, Spain; 5 Laboratory of Experimental Oncology Research, Istituto Ortopedico Rizzoli, Via G.C.Pupilli 1, 40136 Bologna, Italy; 6 Norwegian Microarray Consortium, Institute for Molecular Bioscience, University of Oslo, 0316 Oslo, Norway

## Abstract

**Background:**

Conventional high-grade osteosarcoma is a primary malignant bone tumor, which is most prevalent in adolescence. Survival rates of osteosarcoma patients have not improved significantly in the last 25 years. Aiming to increase this survival rate, a variety of model systems are used to study osteosarcomagenesis and to test new therapeutic agents. Such model systems are typically generated from an osteosarcoma primary tumor, but undergo many changes due to culturing or interactions with a different host species, which may result in differences in gene expression between primary tumor cells, and tumor cells from the model system. We aimed to investigate whether gene expression profiles of osteosarcoma cell lines and xenografts are still comparable to those of the primary tumor.

**Methods:**

We performed genome-wide mRNA expression profiling on osteosarcoma biopsies (n = 76), cell lines (n = 13), and xenografts (n = 18). Osteosarcoma can be subdivided into several histological subtypes, of which osteoblastic, chondroblastic, and fibroblastic osteosarcoma are the most frequent ones. Using nearest shrunken centroids classification, we generated an expression signature that can predict the histological subtype of osteosarcoma biopsies.

**Results:**

The expression signature, which consisted of 24 probes encoding for 22 genes, predicted the histological subtype of osteosarcoma biopsies with a misclassification error of 15%. Histological subtypes of the two osteosarcoma model systems, *i.e*. osteosarcoma cell lines and xenografts, were predicted with similar misclassification error rates (15% and 11%, respectively).

**Conclusions:**

Based on the preservation of mRNA expression profiles that are characteristic for the histological subtype we propose that these model systems are representative for the primary tumor from which they are derived.

## Background

Conventional high-grade osteosarcoma is the most frequent primary malignant bone tumor, with a peak occurrence in children and adolescents and a second peak in patients older than 40 years. It is a highly genetically instable tumor, of which karyotypes often show aneuploidy, high level amplification and deletion, and translocations[1]. No precursor lesion is known, although part of the osteosarcomas in patients over 40 years is secondary, and is induced by radiation, chemicals, or by an underlying history of Paget's disease of bone[2]. The leading cause of death of osteosarcoma patients are distant metastases, which despite aggressive chemotherapy regimens still develop in approximately 45% of all patients[3]. Overall survival of osteosarcoma patients has increased from 10-20% before the introduction of pre-operative chemotherapy in the 1970s, to about 60%[4]. However, survival has reached a plateau, and treating with higher doses of chemotherapy does not lead to better overall survival[5].

Osteosarcoma is a heterogeneous tumor type, which can be subdivided into various subtypes[6]. Conventional high-grade osteosarcoma is the most common subtype, and can be further subdivided in different histological subtypes, of which osteoblastic (50%), chondroblastic (25%), and fibroblastic osteosarcoma (25%) are the most frequent ones. Other subtypes of conventional high-grade osteosarcoma, such as chondromyxoid fibroma-like, clear cell, epitheliod, sclerosing, and giant cell rich osteosarcoma, are extremely rare[2]. Often, osteosarcoma tissue contains a mixture of morphologically differing cell types, and the classification is based on the most dominant type [7]. The three main histological subtypes have different survival profiles. Patients with fibroblastic osteosarcoma have a significantly better response to pre-operative chemotherapy, which is a known predictor for improved survival, than do osteoblastic osteosarcoma patients[8]. Although patients with chondroblastic osteosarcoma are relatively poor responders to pre-operative chemotherapy[7,9], which is probably caused by the impermeability of the chondroid areas of the tumor, there is a trend for these patients to have better 5-year survival profiles[7], but also a higher risk for late relapse[10].

The search for new (targeted) therapies to treat osteosarcoma is ongoing[11]. Because the disease is relatively rare, large efforts need to be done in order to collect a considerable amount of patient samples. Moreover, material is usually scarce due to necrosis in resections of the primary tumor, which is mostly present in tumors of patients who respond fairly well to neo-adjuvant chemotherapy. No necrosis is present in pre-chemotherapy biopsies, but these are often very small and are not readily available for research because they are needed for diagnosis. Because of these limitations, model systems are widely used to study osteosarcomagenesis and for preclinical testing of candidate drugs. Osteosarcoma cell lines, especially SAOS-2 and U-2-OS are frequently used as model systems, remarkably not only to study osteosarcoma, but all types of *in vitro *cell biological processes, as these cell lines grow fast and are relatively easy to transfect. Recently, the EuroBoNeT http://www.eurobonet.eu osteosarcoma panel of 19 cell lines was characterized, which allows us to study osteosarcoma in a high-throughput manner [12]. This panel of osteosarcoma cell lines has been shown to resemble osteosarcoma phenotypically and functionally[13]. Other established model systems include xenografts from primary tumors or osteosarcoma cell lines in immunodeficient nude mice, which subsequently develop into tumors resembling osteosarcoma[13-15]. Osteosarcomagenesis can also be induced in mice by radiation or orthotopically implanting chemical carcinogens[16]. We have previously shown that DNA copy number profiles of xenografts resemble those of the corresponding primary tumor, although some significant changes for osteosarcoma were observed[15].

Established cancer cell lines are often thought not to be representative for the originating primary tumor. Since there could have been a selection for their propensity to grow in culture, they lack interaction with stroma and may have acquired additional mutations in culture[17]. Xenografts do have tumor-host interactions, but can lose matrix as well after several passages. It is not clear whether such changes in matrix composition of xenografts are caused by the tumor cells, or by changes in mouse stroma[14]. Despite these biological differences, model systems are useful for studying signal transduction pathways important in tumor biology, of which mRNA expression, as measured by qPCR or using gene expression microarrays, is frequently used as a readout. It is therefore highly important to determine whether gene expression levels of these model systems are comparable to those of the corresponding primary tumors, which we aimed to do in this study. We performed gene expression analysis on a panel of 76 conventional high-grade osteosarcoma pre-treatment biopsies. We set out to recapitulate representative expression profiles from primary untreated osteosarcoma biopsies in corresponding models *i.e*. cell lines and xenografts. We could demonstrate that both model systems still express genes that are characteristic for the specific histological subtype of the primary tumor. We therefore endorse that, despite biological differences, both xenografts and cell lines are representative model systems for studying mRNA expression in high-grade osteosarcoma. Specific models may be identified that would be appropriate to use for studies of specific subgroups of osteosarcoma.

## Methods

### Ethics statement

All biological material was handled in a coded fashion. Ethical guidelines of the individual European partners were followed and samples and clinical data were stored in the EuroBoNet biobank. For xenograft experiments, informed consent and sample collection were approved by the Ethical Committee of Southern Norway (Project S-06132) and the Institutional Ethical Committee of Valencia University.

### Patient cohorts

Genome-wide expression profiling was performed on pre-treatment diagnostic biopsies of 76 resectable high-grade osteosarcoma patients from the EuroBoNet consortium http://www.eurobonet.eu. Clinicopathological details of these 76 samples can be found in Table 1. Samples with a main histological subtype (n = 66) were selected for subsequent subtype analyses. These 66 samples included 50 osteoblastic, 9 chondroblastic, and 7 fibroblastic osteosarcomas. Five additional osteosarcoma biopsies (1 chondroblastic and 4 osteoblastic osteosarcomas), 12 mesenchymal stem cell (MSC) and 3 osteoblast cultures, and 12 chondrosarcoma biopsies were used for validation.

**Table 1:** Clinicopathological details

Category	Patient characteristics	Biopsies (%)	Cell lines (%)	Xenografts (%)
*Total nr of samples*		76 (100)	13 (100)	18 (100)

*Institution*	LUMC, Netherlands	29 (38.2)	0 (0)	0 (0)

	IOR, Italy	11 (14.5)	7 (53.8)	0 (0)

	LOH, Sweden	3 (3.9)	0 (0)	0 (0)

	Radiumhospitalet, Norway	1 (1.3)	3 (23.1)	12 (66.7)

	UV, Spain	0 (0)	0 (0)	6 (33.3)

	WWUM, Germany	32 (42.1)	0 (0)	0 (0)

	Other	0 (0)	3 (23.1)	0 (0)

*Origin*	Biopsy	76 (100)	0 (0)	0 (0)

	Resection	0 (0)	7 (53.8)	11 (61.1)

	Metastasis	0 (0)	3 (23.1)	1 (5.6)

	Unknown	0 (0)	3 (23.1)	6 (33.3)

*Location of primary tumor*	Femur	36 (47.4)	0 (0)	10 (55.6)

	Tibia/fibula	26 (34.2)	0 (0)	2 (11.1)

	Humerus	10 (13.2)	0 (0)	2 (11.1)

	Axial skeleton	1 (1.3)	0 (0)	1 (5.6)

	Unknown/other	3 (3.9)	13 (100)	3 (16.7)

*Histological subtype*	Osteoblastic	50 (65.8)	9 (69.2)	15 (83.3)

	Chondroblastic	9 (11.8)	0 (0)	3 (16.7)

	Fibroblastic	7 (9.2)	4 (30.8)	0 (0)

	Minor	10 (13.2)	0 (0)	0 (0)

*Histological response to pre-operative chemotherapy in the primary tumor*	Good response	33 (43.4)	0 (0)	0 (0)

	Poor response	36 (47.4)	0 (0)	0 (0)

	Unknown/NA	7 (9.2)	13 (100)	18 (100)

*Sex*	Male	52 (68.4)	9 (69.2)	9 (50)

	Female	24 (31.6)	4 (30.8)	3 (16.7)

	Unknown	0 (0)	0 (0)	6 (33.3)

Clinicopathological details of patients with conventional high-grade osteosarcoma, including all patients from the biopsy, cell line, and xenograft datasets.

### Osteosarcoma cell lines

Out of the EuroBoNeT panel of 19 cell lines, 13 cell lines were recorded to belong to a main histological subtype. This set of 13 cell lines contained 4 cell lines derived from fibroblastic, and 9 cell lines derived from osteoblastic osteosarcomas. The 13 osteosarcoma cell lines IOR/MOS, IOR/OS10, IOR/OS14, IOR/OS15, IOR/OS18, IOR/OS9, IOR/SARG, KPD, MG-63, MHM, OHS, OSA, and ZK-58 were maintained in RPMI 1640 (Invitrogen, Carlsbad, CA, USA) supplemented with 10% fetal calf serum and 1% Penicillin/Streptomycin (Invitrogen) as previously described[12]. Clinical details of the tissue from which these cell lines were derived are shown in Table 1 and are described previously[12].

### Osteosarcoma xenografts

The osteosarcoma xenograft model is described in Kresse *et al*. [15]. In short, human tumors were implanted directly from patient samples and successively passaged subcutaneously in nude mice. Eighteen different xenografts were used, of which 3 were derived from chondroblastic, and 15 from osteoblastic osteosarcomas. Clinical data on primary tumor samples and xenograft passages that were used are shown in Table 1.

### Determination of histological subtypes

Histological subtyping was performed by two pathologists (PCWH, EH) on hematoxylin and eosin (HE) stained slides of all biopsies and of all primary tumors from which the osteosarcoma cell lines and xenografts were derived. Osteoblastic, chondroblastic, and fibroblastic osteosarcoma samples were selected for further study. Other subtypes (anaplastic, chondromyxoid fibroma-like, fibroblastic MFH-like, giant cell rich, pleomorphic, and sclerosing osteosarcoma) were excluded because these subtypes are rare.

### RNA isolation, cDNA synthesis, cRNA amplification, and Illumina Human-6 v2.0 Expression BeadChip hybridization

Osteosarcoma and xenograft tissue was handled as previously described[18]. Osteosarcoma cell lines were prepared as in Ottaviano *et al*. [12] RNA isolation, synthesis of cDNA, cRNA amplification, and hybridization of cRNA onto the Illumina Human-6 v2.0 Expression BeadChips were performed as previously described[18].

### Microarray data pre-processing

Microarray data processing and quality control were performed using the statistical language R[19] as described previously[18]. MIAME-compliant data have been deposited in the GEO database (http://www.ncbi.nlm.nih.gov/geo/, accession number GSE30699). High correlations between these microarray data and corresponding qPCR results have been demonstrated previously[18].

### Detection of significantly differentially expressed genes

We performed *LIMMA *analyses[20] in order to determine differential expression for the following clinical parameters: sex (52 male *vs *24 female), tumor location (36 femur, 10 humerus, 26 fibula/tibia), response to pre-operative chemotherapy (36 poor responders, or Huvos grade 1-2, *vs *33 good responders, or Huvos grade 3-4), and histological subtype (a factorial analysis comparing 50 osteoblastic, 9 chondroblastic, and 7 fibroblastic osteosarcomas). Genes that play a role in metastasis-free survival are described in Buddingh *et al*. [18]. Probes with Benjamini and Hochberg False discovery rate-adjusted *P*-values (adj*P*) < 0.05 were considered to be significantly differentially expressed.

### Prediction analysis

The gene expression profile was generated on the dataset of biopsies using Bioconductor[21] package *PAMR*[22]. Internal cross-validation was performed 50 times. A threshold was selected where the error rate of the prediction profile was minimal. The minimum error rate was representative of 50 independent simulations. In order to minimize optimization bias[23], we validated the profile on an independent dataset of osteosarcoma biopsies (n = 5), containing 1 chondroblastic osteosarcoma and 4 osteoblastic osteosarcomas. In addition, we applied the profile on datasets containing positive controls - mesenchymal stem cells (MSC, n = 12), osteoblasts (n = 3), and chondrosarcoma biopsies (n = 12, previously published in[24], GEO accession number GSE12532). We subsequently applied the validated prediction profile to two independent datasets, the first consisting of gene expression data of osteosarcoma cell lines, the second of xenografts. Expression of the probes that composed the prediction profile was verified using a factorial *LIMMA *analysis, comparing chondroblastic, fibroblastic, and osteoblastic osteosarcoma biopsy samples.

### Gene set enrichment

Network analysis was performed using Ingenuity Pathways Analysis (IPA, Ingenuity Systems, http://www.ingenuity.com). For both chondroblastic-specific and fibroblastic-specific analyses, data for all reference sequences containing expression values and FDR-adjusted *P*-values were uploaded into the application. Each identifier was mapped to its corresponding object in Ingenuity's Knowledge Base. An adj*P *cut-off of 0.05 was set to select genes whose expression was significantly differentially regulated. The Network Eligible molecules were overlaid onto a global molecular network developed from information contained in Ingenuity's Knowledge Base. Networks of Network Eligible Molecules were then algorithmically generated based on their connectivity. GO term enrichment was tested using Bioconductor package *topGO*[25]. Lists of significantly affected genes were compared with all genes eligible for the analysis. GO terms with Fisher's exact *P*-values < 0.001, as calculated by the *weight *algorithm from *topGO*, were defined significant.

## Results

### Histological subtypes of osteosarcoma biopsies have different gene expression profiles

**Figure 1:**
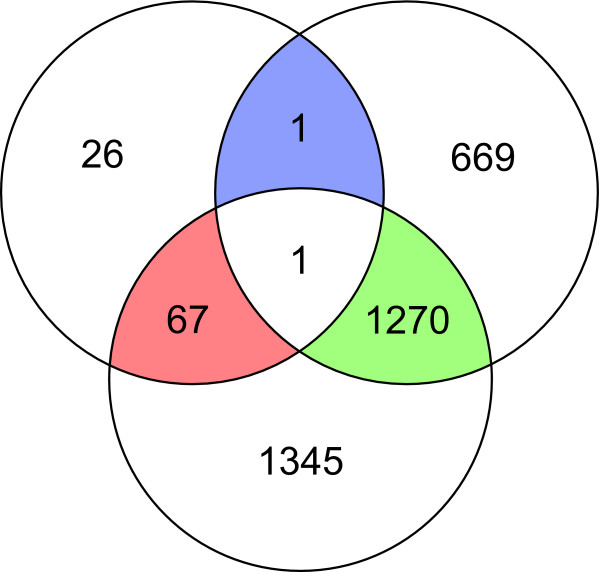
**Subtype-specific genes**. Venn diagram representing numbers of fibroblastic- (green), chondroblastic- (red), and osteoblastic (blue)-specific differentially expressed genes obtained with factorial *LIMMA *analysis, considering chondroblastic versus osteoblastic (chondro *vs *osteo), fibroblastic versus osteoblastic (fibro *vs *osteo), and chondroblastic versus fibroblastic (chondro *vs *fibro) analyses. Subtype-specific genes are genes that are either both upregulated or both downregulated in the subtype of interest in the different comparisons.

We determined differential expression for different clinical parameters. Of all comparisons of clinical parameters only histological subtypes appeared to give a sufficient number of differentially expressed genes to design a prediction profile. *LIMMA *analyses resulted in one location-specific differentially expressed gene: *HOXD4*, which was overexpressed in tumors at the humerus versus at fibula/tibia and femur. Between tumors from male and female patients, 18 genes were significantly differentially expressed, all belonging to X- and Y-chromosome-specific genes, which are not considered as representative for osteosarcoma, yet this finding validates the analysis. No significantly affected genes were returned with regards to response to pre-operative chemotherapy. To determine differential expression between the three main histological subtypes, we excluded all samples with unknown or rare subtypes. This resulted in a dataset of 66 conventional high-grade osteosarcoma biopsies with a main histological subtype. Using a factorial *LIMMA *analysis, we determined 1338 significantly differentially expressed genes (adj*P *< 0.05) that were specific for a certain main histological subtype (depicted in a Venn diagram in Figure 1). A subtype-specific probe was defined as a probe that had the same sign of log fold change in both analyses, *e.g*. the probe was upregulated in chondroblastic samples versus osteoblastic, and in chondroblastic versus fibroblastic samples.

### Gene set enrichment shows specific sets of genes are affected in fibroblastic and chondroblastic osteosarcoma

Network analysis using IPA showed that fibroblastic osteosarcoma-specific networks mostly had a role in cellular growth and proliferation, which was also the most significant biological function as detected by IPA (see Additional File 1 for all affected networks and biological functions). The most significant network is illustrated in Additional File 2A and shows that mRNA of various genes with a connection to the NF-κB pathway and STAT5A signaling are upregulated in fibroblastic osteosarcoma biopsies, as compared with both osteoblastic and chondroblastic osteosarcoma. The most significant network specific for the chondroblastic subtype consisted of genes important in skeletal connective tissue development and function (Additional File 2B), and shows that, also on the gene expression level, chondroblastic osteosarcoma is mainly distinguished from osteoblastic and fibroblastic osteosarcoma based on the composition of the extracellular matrix of the tumor (Additional File 1 shows all affected networks and biological functions).

Results from network analysis were confirmed using *topGO*, a gene set enrichment approach analyzing the significance of GO terms in a specific dataset. These analyses resulted in two significant fibroblastic specific GO terms in osteosarcoma: regulation of tyrosine phosphorylation of Stat5 protein (GO:0042522, *P *= 4.8E-4) and regulation of survival gene product expression (GO:0045884, *P *= 8.2E-4). Significantly differentially expressed genes from both GO terms partly overlap the fibroblastic osteosarcoma-specific network detected with IPA. Two GO terms were significant in the chondroblastic-specific analysis as well: skeletal system development (GO:0001501), and extracellular matrix organization (GO:0030198), which strengthen the results found in the IPA network analyses. GO term subgraphs of the five most significant GO terms for both analyses are shown in Additional File 3.

Gene set enrichment on genes specific for osteoblastic osteosarcoma was not performed, because only one osteoblastic osteosarcoma-specific probe was detected that distinguishes the osteoblastic subtype from fibroblastic and chondroblastic. This probe matches to *UNQ1940*, or *FAM180A*, a protein-coding gene with a yet unknown function.

### Generation and validation of the prediction profile

**Figure 2:**
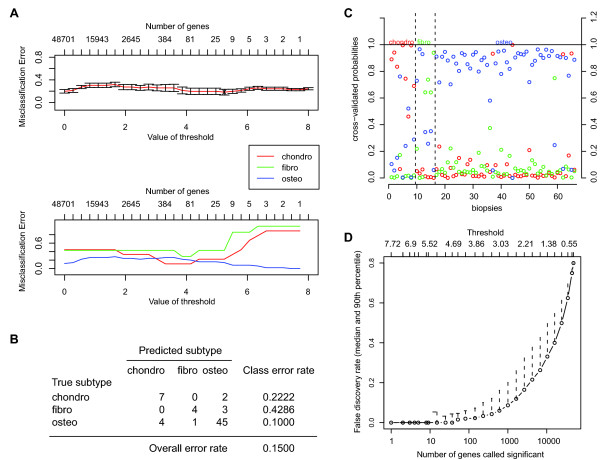
**Nearest shrunken centroids classification**. *A *Illustration of training the *pamr *prediction profile on osteosarcoma biopsies. At thresholds of 4.9-5.1, the misclassification error rate was minimal. *B *True versus predicted values from the nearest shrunken centroid fit. *C *Probabilities of each biopsy to belong to any of the three histological subtypes. Samples are separated (dotted lines) based on their true subtypes. Cross-validated probabilities for each histological subtype are shown on the y-axis, so that for every sample three open dots are present (blue, red, and green dots for osteo-, chondro-, and fibroblastic osteosarcoma, respectively). A sample is classified into a specific subtype if the probability to belong to that specific subtype is higher than the probabilities to belong to the other subtypes. *D *The FDR plotted against different thresholds of the prediction profile. At a threshold of 5.0, 24 genes are included in the prediction profile. These 24 genes have a FDR < 5%.

Because we could not directly compare subtype-specific genes between our different model systems due to small sample sizes, we generated a profile that could predict the histological subtype of osteosarcoma. The prediction profile was generated on 66 high-grade conventional osteosarcoma pre-chemotherapy biopsies, using nearest shrunken centroids classification. Optimal control of error rate in the prediction profile was found at delta thresholds of 4.9-5.1 (Figure 2A), where merely 10 out of 66 samples (15%) in the training set were wrongly assigned to a specific histological subtype. This error rate was representative for a set of 50 simulations, which resulted in error rates between 13.5% and 15%. Subtype-specific error rates were 22%, 43%, and 10% for chondroblastic, fibroblastic, and osteoblastic subtypes, respectively (Figure 2B). Probabilities of each sample to belong to any of the three histological subtypes are shown in Figure 2C. At a threshold delta of 5.0, the prediction profile consisted of 24 probes encoding for 22 genes. All genes were below a FDR threshold of 5% (Figure 2D). Expression of the 24 probes of the profile were verified in a factorial *LIMMA *analysis which was corrected for multiple testing. All 24 probes were confirmed to be significantly differentially expressed in the *LIMMA *analysis as well. Results from *pamr *and *LIMMA *analyses are shown in Table 2. A supervised heatmap depicting expression of the 24 probes in all samples is shown in Additional File 4. The prediction profile was validated at threshold delta of 5.0 in an independent dataset of osteosarcoma biopsies and positive controls. Histological subtypes of biopsies had a prediction error of 0% (0/5). Mesenchymal stem cells and osteoblasts all fitted in the osteoblastic group, while 11/12 chondrosarcoma samples were best corresponding to the group of chondroblastic osteosarcoma. The remaining chondrosarcoma sample was a dedifferentiated chondrosarcoma and was predicted in the fibroblastic group, probably because of the high amount of spindle cells present in the biopsy. Additional File 5 shows prediction probabilities for each subtype of these additional datasets.

**Table 2:** Comparison of the prediction profile with *LIMMA *analysis

probeID	symbol	*LIMMA *logFC C*vs*F	*LIMMA *logFC C*vs*O	*LIMMA *logFC F*vs*O	*LIMMA *adj*P*	*pamr *chondro-score	*pamr *fibro-score	*pamr *osteo-score
5910377	*ACAN*	2.42	2.24	-0.18	0.0000	0.9294	0	-0.0147

3390678	*NFE2L3*	-1.74	-0.02	1.71	0.0000	0	0.9184	0

1990523	*COL9A1*	3.49	3.01	-0.47	0.0000	0.6011	0	0

360553	*SCRG1*	4.55	3.51	-1.04	0.0001	0.4571	0	0

3310368	*ID3*	1.87	-0.29	-2.16	0.0003	0	-0.4053	0

10561	*ITIH5L*	0.68	0.65	-0.04	0.0001	0.295	0	0

5050110	*MGC34761*	0.93	0.83	-0.09	0.0002	0.2818	0	0

4780368	*ACAN*	1.34	1.19	-0.14	0.0004	0.2716	0	0

7150719	*COL2A1*	4.82	4.36	-0.47	0.0016	0.183	0	0

3830341	*LYRM1*	-1.23	-0.18	1.06	0.0007	0	0.1677	0

3990500	*MATN4*	1.96	1.69	-0.27	0.0012	0.151	0	0

4280370	*POPDC3*	-0.88	-0.08	0.80	0.0009	0	0.0909	0

6520487	*UNQ830*	4.10	2.90	-1.20	0.0016	0.0817	0	0

2850202	*COL11A2*	1.37	1.10	-0.27	0.0014	0.0735	0	0

4220452	*C11ORF41*	-0.89	-0.03	0.86	0.0011	0	0.0721	0

4560091	*COL9A3*	1.14	1.21	0.07	0.0018	0.0698	0	0

5890452	*LOC652881*	0.43	0.37	-0.06	0.0001	0.0666	0	0

3990259	*PPP2R2B*	-1.00	0.10	1.10	0.0016	0	0.0603	0

5340392	*MAN2A1*	-1.42	-0.22	1.20	0.0018	0	0.0477	0

3360139	*DLX5*	1.84	-0.20	-2.04	0.0033	0	-0.0358	0

2630762	*C14ORF78*	-1.07	1.45	2.52	0.0011	0	0	-0.0307

3460037	*UNQ1940*	0.44	1.71	1.27	0.0018	0	0	-0.0219

6110722	*COL2A1*	1.22	1.44	0.22	0.0032	0.0087	0	0

6980164	*ALPL*	2.52	-0.67	-3.19	0.0038	0	-0.0036	0

Comparison of the 24 genes obtained with *pamr *prediction with a factorial *LIMMA *analysis between the three different histological subtypes (C*vs*F: chondroblastic- *vs *fibroblastic, C*vs*O: chondroblastic- *vs *osteoblastic, F*vs*O: fibroblastic *vs *osteoblastic osteosarcoma), for which log fold changes (logFC) are shown for the different coefficients of the analysis. Note that the adj*P *shows the significance for the whole factorial *LIMMA *analysis, and does not reflect the adj*P*s per subanalysis.

### A prediction profile based on osteosarcoma biopsy data can predict histological subtypes of cell lines and xenografts

**Figure 3:**
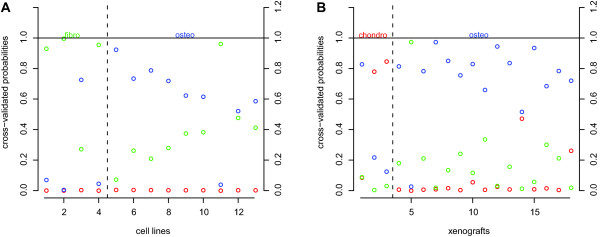
**The prediction profile applied on cell lines and xenografts**. Probabilities of *A *cell lines and *B *xenografts to belong to any of the three histological subtypes. For an explanation of what is represented by these graphs, see Figure 2C.

Unsupervised clustering of all biopsies, xenografts, and cell lines demonstrated that xenografts and cell lines show different overall expression profiles from most biopsies, and that there are no subtype-specific clusters based on overall expression (Additional File 6). In order to determine whether histological subtypes of cell lines and xenografts could be predicted as well with the 24-gene prediction profile, we applied this profile to two independent datasets. In the first dataset, consisting of osteosarcoma cell line data, 2 out of 13 samples (15%, Figure 3A) were wrongly classified. These samples were MG63, a cell line derived from a fibroblastic osteosarcoma, which was subtyped as being osteoblastic, and IOR/OS18, derived from an osteoblastic osteosarcoma, which was subtyped by the prediction profile as a fibroblastic osteosarcoma. Interestingly, HOS, HOS-MNNG, and HOS-143B, all cell lines derived from the HOS cell line, which is derived from fibroblastic and epithelial osteosarcoma and therefore was not added to our set of 13 osteosarcoma cell lines, were all predicted as fibroblastic osteosarcoma (data not shown). Two out of 18 xenograft samples (11%, Figure 3B) were wrongly classified. One of these samples was OKx, a xenograft derived from a chondroblastic tumor, which was subtyped as an osteoblastic osteosarcoma. The other sample was KPDx, a xenograft derived from an osteoblastic tumor, which was subtyped as fibroblastic. The KPD cell line was subtyped rightly as an osteoblastic osteosarcoma.

## Discussion

In this study, we aimed to compare gene expression profiles of osteosarcoma biopsies with cell lines and xenografts, in order to investigate whether these model systems are representative for the primary tumor. We have determined differential gene expression for different clinical parameters important in high-grade osteosarcoma on a dataset consisting of 76 conventional high-grade osteosarcoma samples. Importantly, pre-treatment biopsies were used instead of resected specimens, because pre-operative chemotherapy may cause tumor necrosis in responsive patients, thus altering gene expression and hampering the generation of high quality mRNA. We intended to generate a gene expression profile that could not only predict a specific clinical parameter in biopsies, but in osteosarcoma cell lines and xenografts as well. The metastasis/survival profile is described previously and may serve as a tool to predict prognosis and as a target for therapy[18]. However, since most of the genes associated with osteosarcoma metastasis were macrophage associated, and no stroma or infiltrate is present in cell lines, this profile could not be applied to osteosarcoma cell lines. We therefore compared gene expression profiles of these different sample sets based on other clinical parameters. No significant differentially expressed genes were found between poor and good responders to chemotherapy. Several reports on genome-wide expression profiling in osteosarcoma have been published describing detection of differential expression between poor and good responders of pre-operative chemotherapy[26-29]. However, the cohorts used in these studies are all relatively small (n = 13-30), and, more importantly, the reported *P*-values were not corrected for multiple-testing in these studies. Remarkably, only two of the genes that were found to correlate with response to chemotherapy in these studies overlap, and one of these two genes was upregulated in poor responders in one study, whereas it was upregulated in good responders in the other study[26,29]. Another report described differential expression between a metastatic and a non-metastatic cell line, for which metastatic capacity correlates with response to chemotherapy[30]. In that particular study, four genes out of 252 were found to overlap with a patient study by Mintz *et al*. [26]. However, the up- and downregulation of these four genes were not consistent between the two studies. We clearly show in a large cohort that there are no differences between these groups of patients, as the most significant probe had an adj*P *of 0.9998. These results are in line with our previous findings obtained by analyzing an osteosarcoma cohort on a different platform[31]. The parameter 'histological subtypes' resulted in a considerable number of differentially expressed genes. Our prediction profile is not directly applicable to other platforms, but there is no real need to have a prediction profile for primary osteosarcoma histological subtype, since pathologists are very well able to assess this on an HE-section, even on a biopsy, with a concordance of 98% between histological subtype of biopsies and corresponding resections[7]. Yet, we here show a quite important use of this profile, *i.e*. to determine the histological subtype of cell lines and xenografts. *In vitro *2-dimensional growing cells lack extra cellular matrix formation, which is the characteristic feature to distinguish histological subtypes in high-grade central osteosarcoma.

The gene expression profiles as detected by analyzing osteosarcoma biopsy data show a large number of subtype-specific differentially expressed genes. In particular, fibroblastic osteosarcoma differed most from the two other main subtypes. Using gene set enrichment, we detected a network of genes upregulated in fibroblastic osteosarcoma, with a role in cellular growth and proliferation, and connection to the NF-κB pathway. This may be a readout of the high cellularity and low matrix composition of fibroblastic osteosarcoma in comparison with osteoblastic and chondroblastic osteosarcoma[32]. GO term enrichment analysis confirmed these results. These pathways may explain why it is comparatively easy to culture fibroblastic osteosarcoma cells, which also may explain why the percentage of fibroblastic osteosarcoma is relatively high in our cell line dataset (31%, compared to 11% in the biopsy dataset). Next to this link to cellular growth and proliferation, the most significant network with fibroblastic-specific upregulated genes showed a connection to the immune system. GO analysis of the five most significant GO terms pointed to involvement of the immune system as well (GO term GO:0006955, *P *= 3.9E-3, see Additional File 3 for GO term subgraphs). Forty-four genes in this GO term were significant, of which 43 were upregulated in fibroblastic osteosarcoma. An elevated immune response might be the reason why patients with fibroblastic osteosarcoma tend to have better survival profiles, as a pro-inflammatory environment has an important role in osteosarcoma metastasis-free survival. This profile is different from the previously found macrophage-specific profile which was associated with better metastasis-free survival of osteosarcoma patients[18]. The overrepresentation of pathways involved in chondrogenesis in the chondroblastic profile is in line with the high amount of chondroid matrix in this subtype. We only detected one osteoblastic-specific gene, *UNQ1940*, or *FAM180A*, with a yet unknown function. Since 50 osteoblastic osteosarcoma samples were compared with only 9 chondroblastic and 7 fibroblastic osteosarcoma samples, we suggest that fibroblastic and chondroblastic osteosarcoma have specific characteristics that distinguishes these tumors from osteoblastic osteosarcoma, and that the latter does not have such an extra feature in comparison with chondro- and fibroblastic osteosarcoma.

Our histological subtype prediction profile consists of 24 probes encoding for 22 genes, all with a specific score which depends on the significance of each gene. The genes that make up the chondroblastic-specific part of this expression profile are mostly chondroid matrix-associated genes, such as *ACAN*, *COL2A1*, and *MATN4*, and are all upregulated in chondroblastic osteosarcoma. Fibroblastic-specific genes that make up the prediction profile are up- or downregulated. An example of a gene upregulated in fibroblastic osteosarcoma is *NFE2L3*, a transcription factor which heterodimerizes with small musculoaponeurotic fibrosarcoma factors and for which a protective role was suggested in hematopoietic malignancies[33]. *DLX5*, a transcription factor involved in bone formation, is downregulated in fibroblastic osteosarcoma, and reflects the lower amounts of matrix present in fibroblastic osteosarcoma. No known function is yet available for the two osteoblastic-specific genes. The misclassification error of the prediction profile in the training set of biopsies was 15%. Cell lines and xenografts were predicted with misclassification errors of 15% and of 11%, respectively. It seems most likely that these prediction errors are inherent to the error rate of the prediction profile, which is also 15%. Thus, because these misclassification errors are in the same range, we suggest that gene expression of these model systems is highly similar to gene expression of the tumor from which they are derived. This is especially of interest for studies in cell lines, since no stroma is present on which subtyping can be performed, but repeatedly passaged xenografts often lose stroma as well. Most genes of the prediction profile are matrix-associated genes. Even though these cell lines do not secrete any matrix, and xenografts have diminished amounts of matrix, we can still detect histological subtype markers on an mRNA level, and are able to distinguish different histological subtypes of cell lines and xenografts using this profile.

## Conclusions

As osteosarcoma xenografts and cell lines still express histological subtype-specific mRNAs that are characteristic of the primary tumor, these model systems are representative for the primary tumor from which they are derived, and will be useful tools for studying mRNA expression and pathways important in high-grade osteosarcoma.

## Competing interests

The authors declare that they have no competing interests.

## Authors' contributions

MLK performed all bioinformatics analyses and drafted the manuscript. MLK, HMN, and IM collected clinical data. IM and ALB provided and prepared xenograft material. SHK and MS provided and prepared cell lines. EIH and PCWH histologically reviewed all samples. MLK, LAMZ, OM, and AMCJ conceived and designed the experiments. All authors read and approved the final manuscript.

## Pre-publication history

The pre-publication history for this paper can be accessed here:

http://www.biomedcentral.com/1755-8794/4/66/prepub

## Supplementary Material

Additional file 1 **List of subtype-specific networks and biological functions**. Networks and biological functions as returned by IPA for fibroblastic- and chondroblastic-specific lists of differentially expressed genes. *P*-values for biological functions are BH FDR-corrected. Click here for file

Additional file 2 **Subtype-specific networks**. *A *Top fibroblastic-specific IPA network showing upregulation of genes connected with NF-κB. *B *Top chondroblastic-specific network illustrating the importance of chondroid-matrix in these samples. Click here for file

Additional file 3 **Subtype-specific GO term subgraphs**. GO term subgraphs of the 5 most significant GO terms for *A *fibroblastic- and *B *chondroblastic-specific genes. GO term subgraphs were generated using Bioconductor package *topGO*. Click here for file

Additional file 4 **Heatmap depicting expression levels of probes in the prediction profile**. A supervised heatmap was generated using R function *heatmap *from the R package *stats*. In the heatmap, low to high probe expression is shown from blue to yellow. The bars above and to the immediate left of the heatmap show whether samples are of the chondroblastic (red), fibroblastic (green), or of the osteoblastic (blue) subtype. The upper bar represents whether samples are biopsies (black), xenografts (magenta), or cell lines (cyan). The outer left bar depicts the regulation of a specific gene in the specific subtype, with yellow for overexpression and blue for downregulation. For the genes *ACAN *and *COL2A1*, two probes are present in the prediction profile. These are indicated as ACAN_1, ACAN_2, COL2A1_1, and COL2A1_2 (probes 4780368, 5910377, 6110722, and 7150719, respectively). Click here for file

Additional file 5 **Validation of the prediction profile**. Predictions of *A *an additional set of biopsies and of the control samples *B *MSCs, *C *osteoblasts, and *D*, chondrosarcoma biopsies to resemble either of the three histological subtypes. For an explanation of what is represented by these graphs, see Figure 2C. Click here for file

Additional file 6 **Dendrogram of osteosarcoma biopsies, xenografts, and cell lines**. Hierarchical unsupervised clustering on all biopsies, xenografts, and cell lines was performed with R function *hclust *from the R package *stats*, using the Euclidian distance, and 1/10^th ^of all probes with the highest variation. We used the Radial Cladogram option in the software Dendroscope http://www.dendroscope.org to visualize the results. *A *Distribution of the different sample types, *B *distribution of the different histological subtypes. Click here for file
