# Supplementary figures and images for: mRNA expression profiles of primary high-grade central osteosarcoma are preserved in cell lines and xenografts
Source: BMC Med Genomics. 2011 Sep 20;4:66. doi: 10.1186/1755-8794-4-66 (PMC3193807; doi:10.1186/1755-8794-4-66)

A

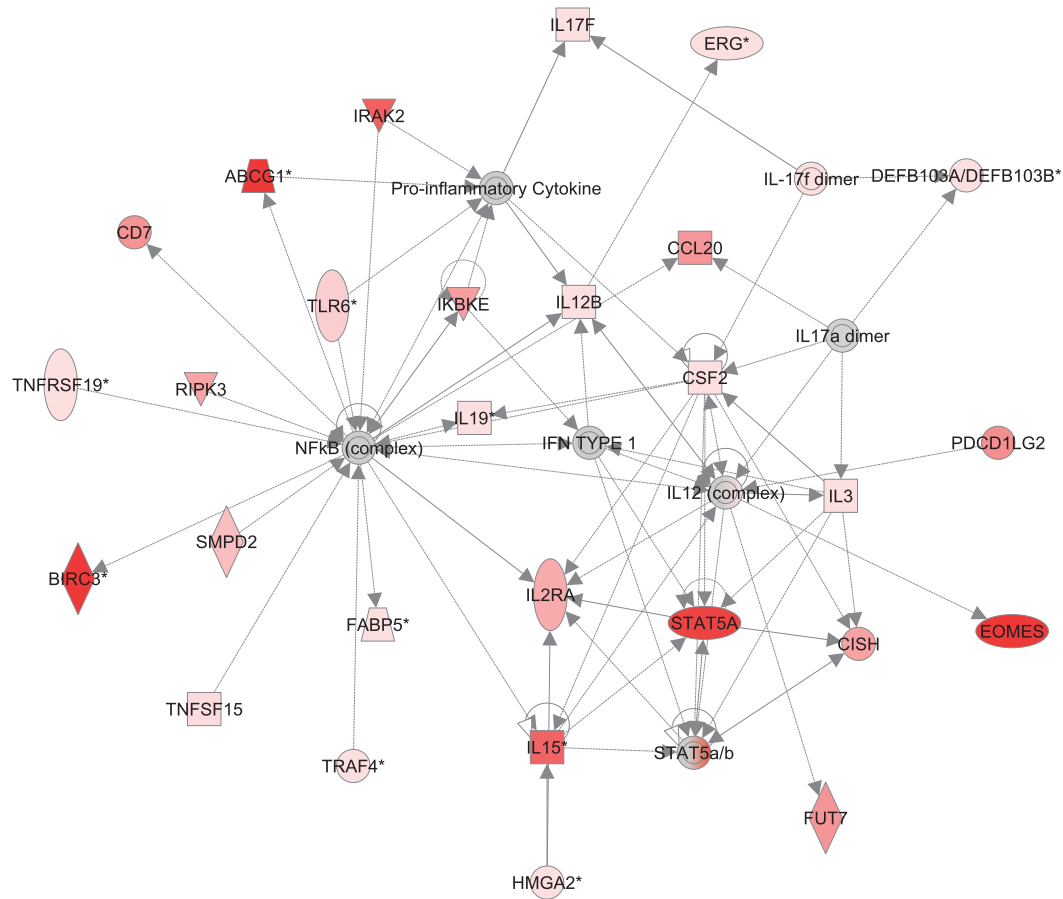

B

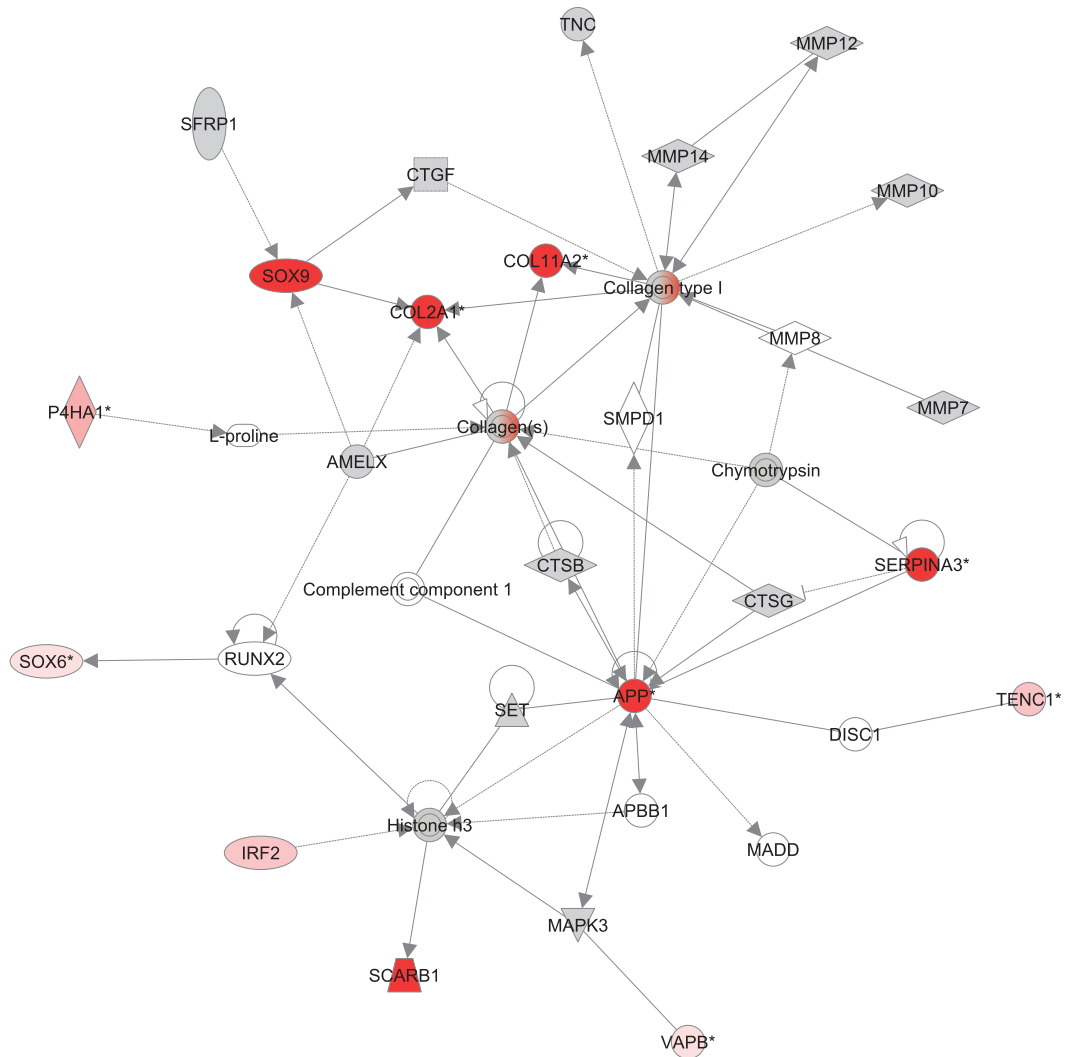

Supplement: Additional file 2 — Subtype-specific networks. A Top fibroblastic-specific IPA network showing upregulation of genes connected with NF-κB. B Top chondroblastic-specific network illustrating the importance of chondroid-matrix in these samples. [file 1755-8794-4-66-S2.PDF]

A

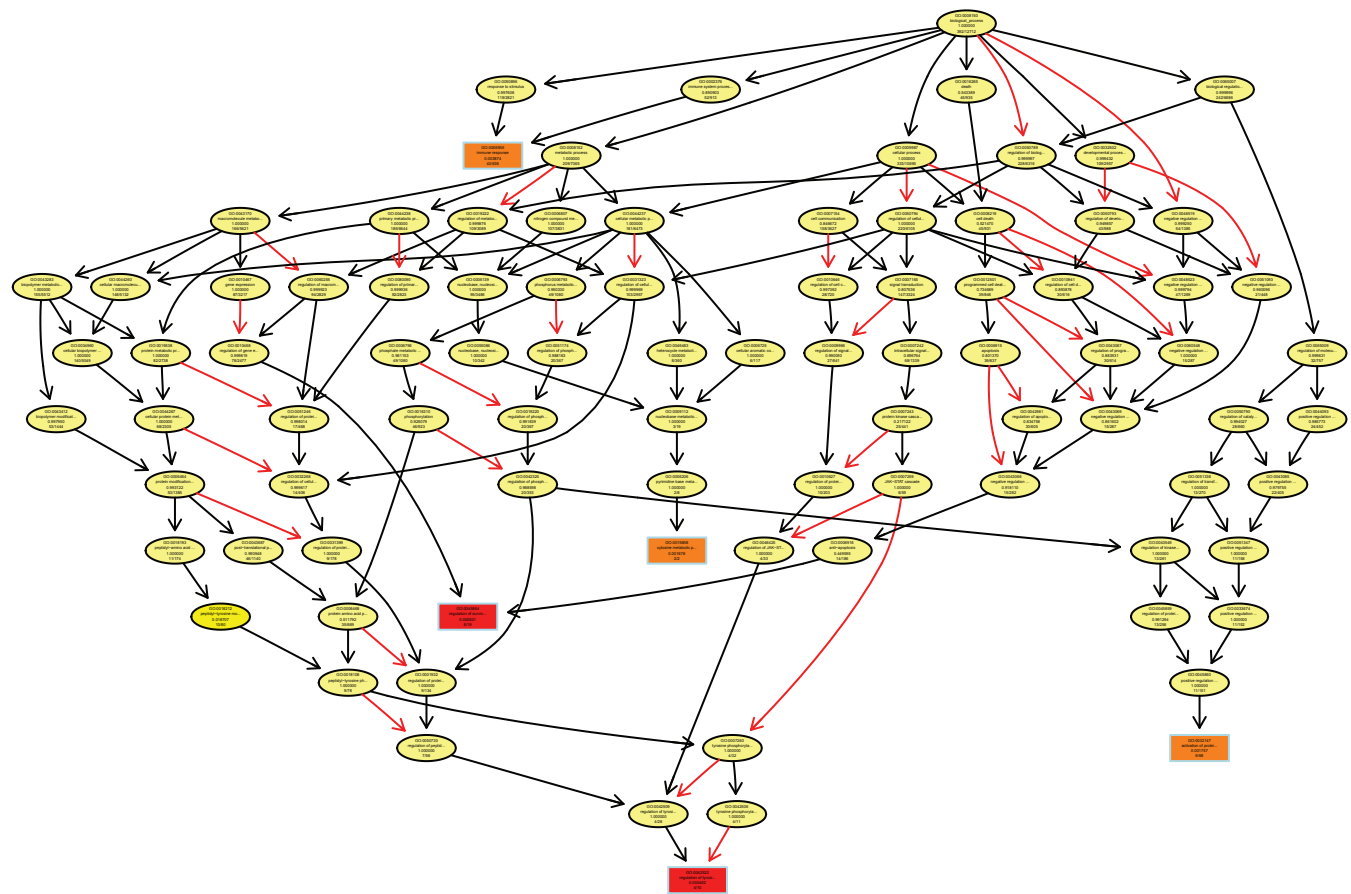

B

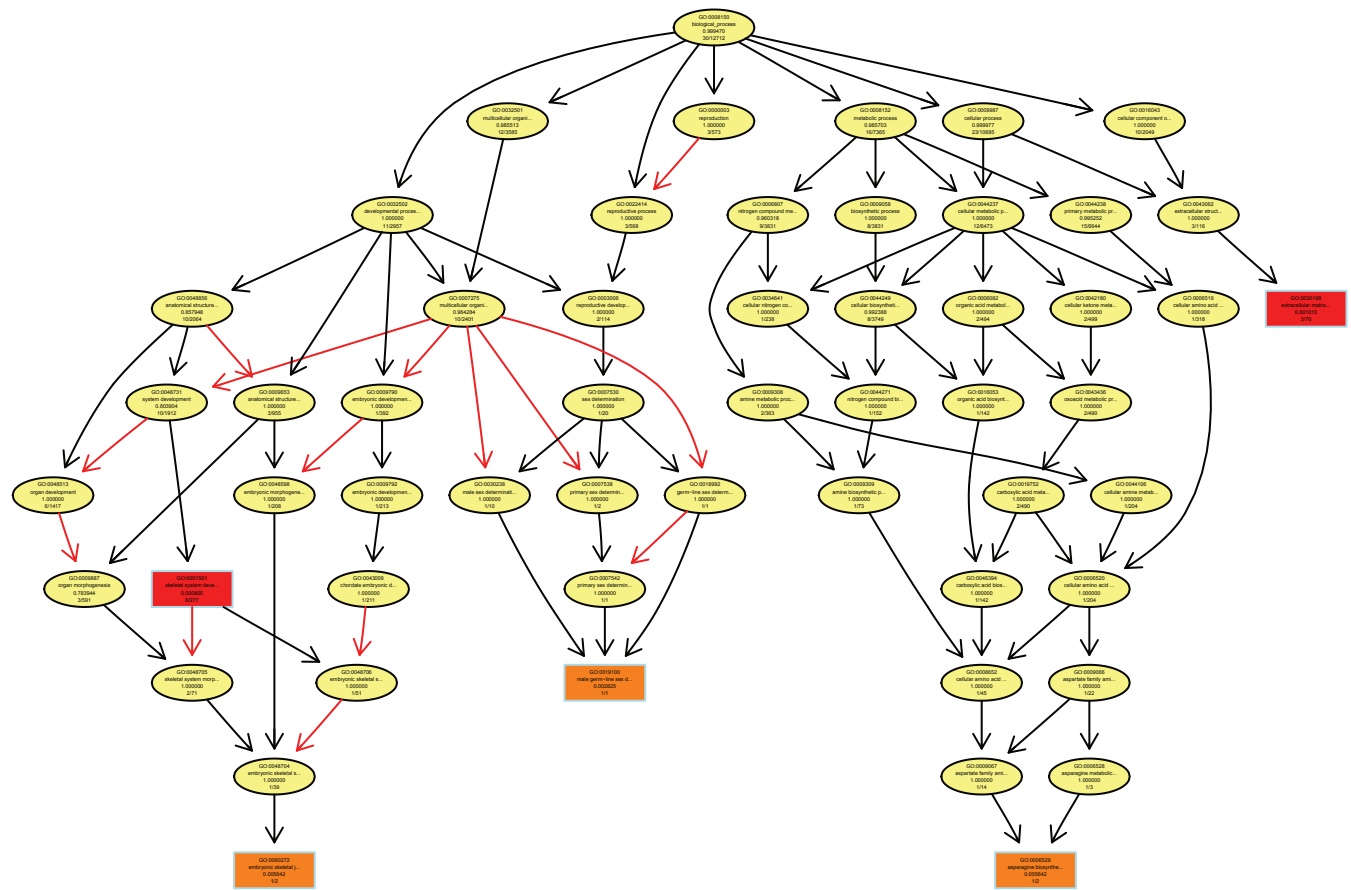

Supplement: Additional file 3 — Subtype-specific GO term subgraphs. GO term subgraphs of the 5 most significant GO terms for A fibroblastic- and B chondroblastic-specific genes. GO term subgraphs were generated using Bioconductor package topGO. [file 1755-8794-4-66-S3.PDF]

chondro, fibro, osteo  
up-, downregulation

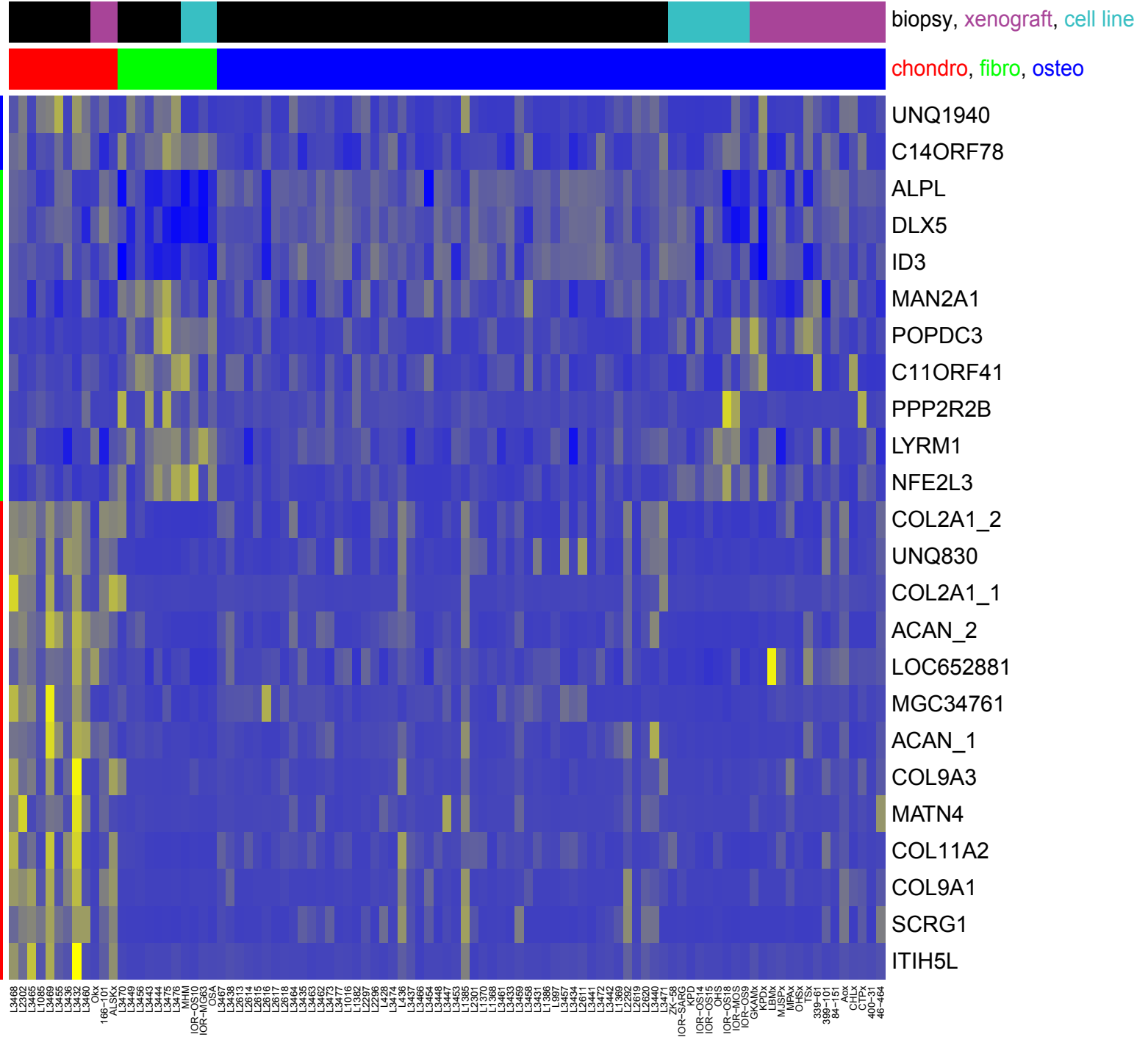

Supplement: Additional file 4 — Heatmap depicting expression levels of probes in the prediction profile. A supervised heatmap was generated using R function heatmap from the R package stats. In the heatmap, low to high probe expression is shown from blue to yellow. The bars above and to the immediate left of the heatmap show whether samples are of the chondroblastic (red), fibroblastic (green), or of the osteoblastic (blue) subtype. The upper bar represents whether samples are biopsies (black), xenografts (magenta), or cell lines (cyan). The outer left bar depicts the regulation of a specific gene in the specific subtype, with yellow for overexpression and blue for downregulation. For the genes ACAN and COL2A1, two probes are present in the prediction profile. These are indicated as ACAN_1, ACAN_2, COL2A1_1, and COL2A1_2 (probes 4780368, 5910377, 6110722, and 7150719, respectively). [file 1755-8794-4-66-S4.PDF]

**A**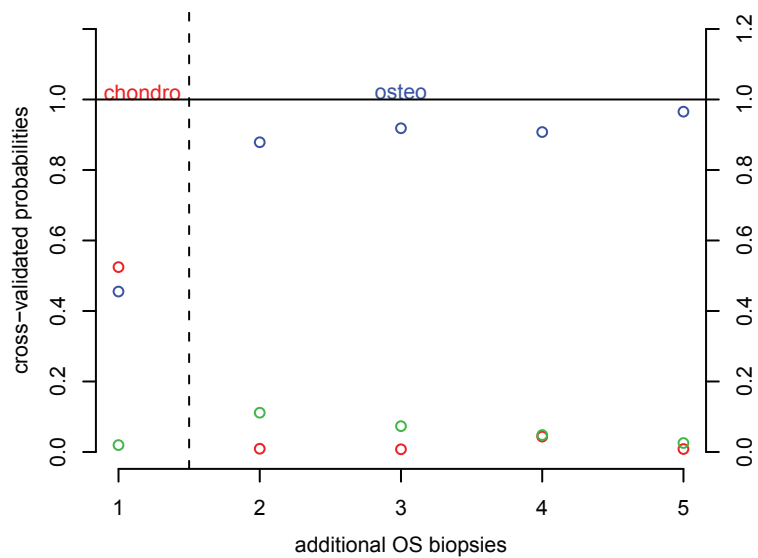**B**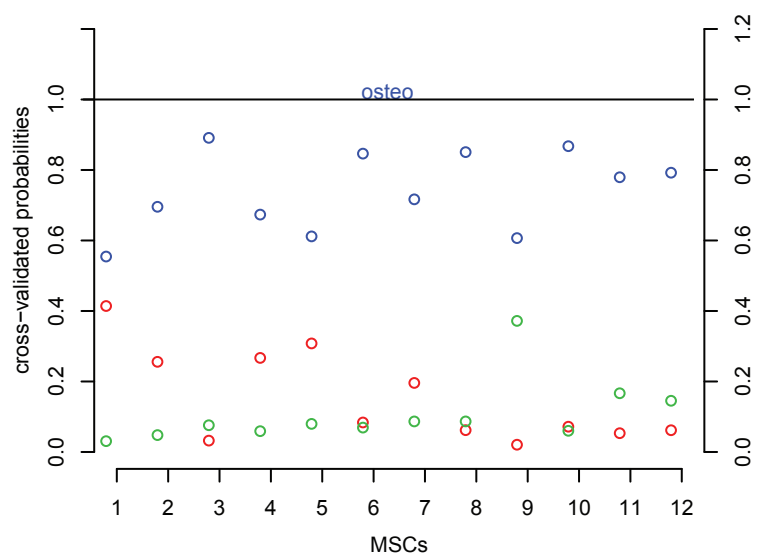**C**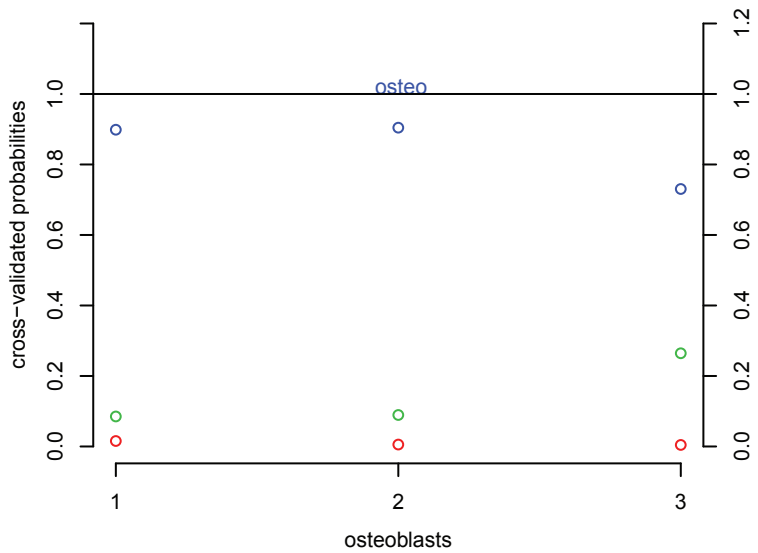**D**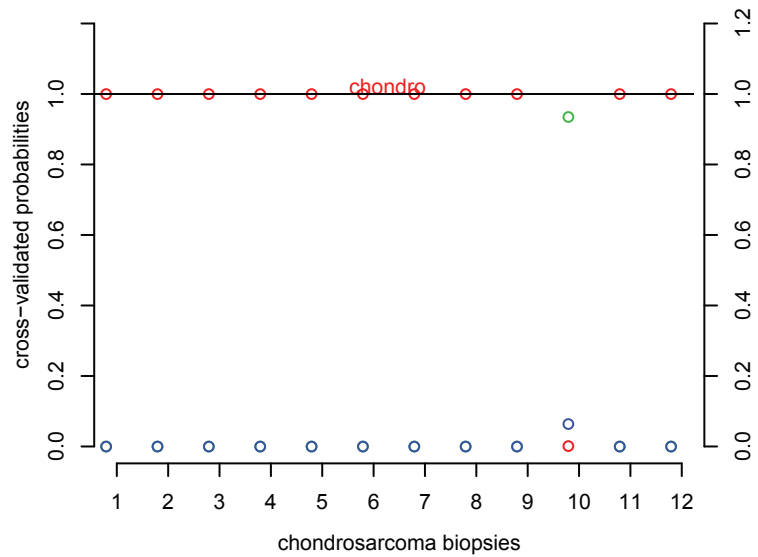

Supplement: Additional file 5 — Validation of the prediction profile. Predictions of A an additional set of biopsies and of the control samples B MSCs, C osteoblasts, and D, chondrosarcoma biopsies to resemble either of the three histological subtypes. For an explanation of what is represented by these graphs, see Figure 2C. [file 1755-8794-4-66-S5.PDF]

A

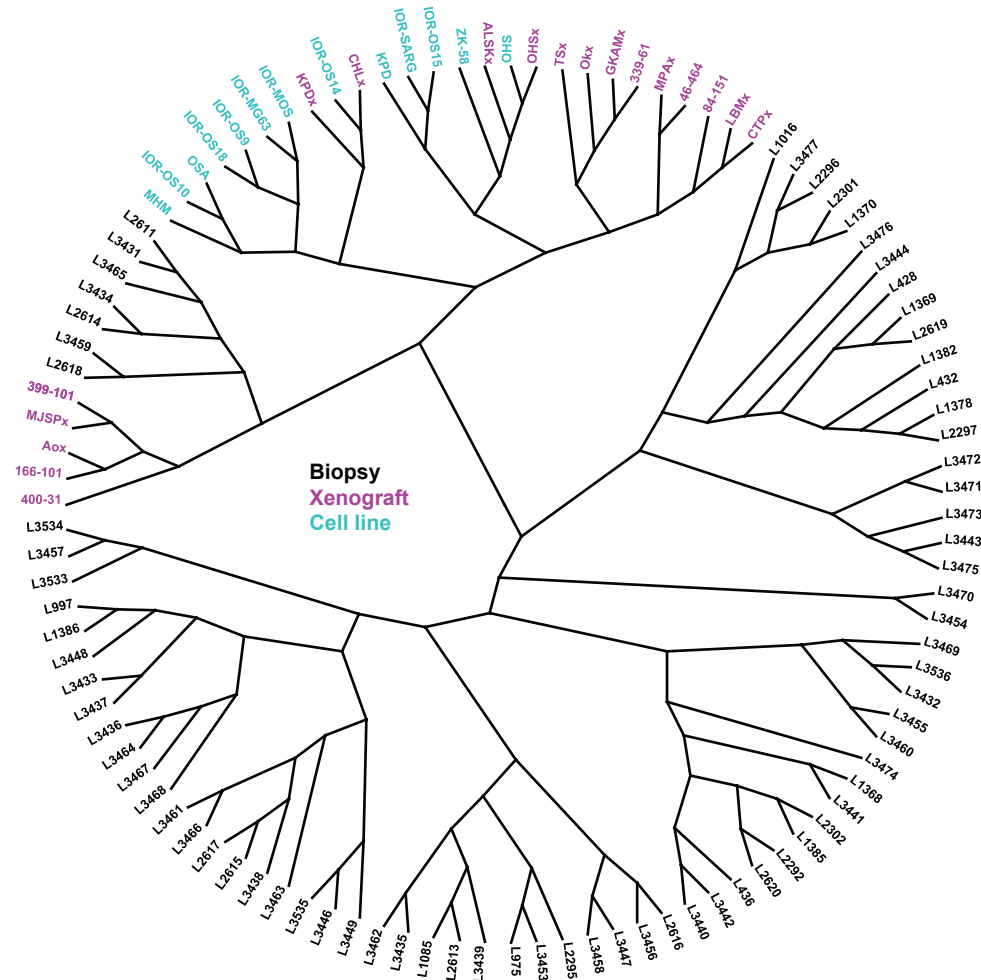

B

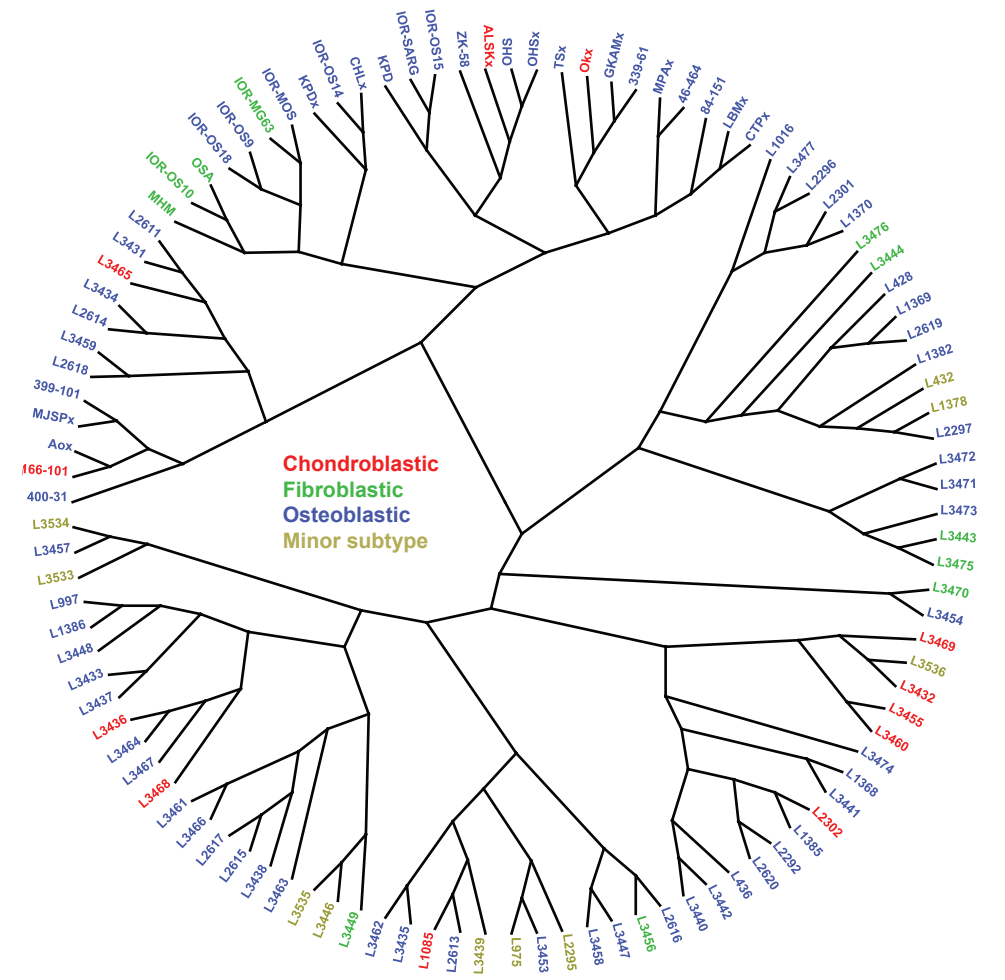

Supplement: Additional file 6 — Dendrogram of osteosarcoma biopsies, xenografts, and cell lines. Hierarchical unsupervised clustering on all biopsies, xenografts, and cell lines was performed with R function hclust from the R package stats, using the Euclidian distance, and 1/10th of all probes with the highest variation. We used the Radial Cladogram option in the software Dendroscope http://www.dendroscope.org to visualize the results. A Distribution of the different sample types, B distribution of the different histological subtypes. [file 1755-8794-4-66-S6.PDF]
